# Placental cytochrome P450 methylomes in infants exposed to prenatal opioids: exploring the effects of neonatal opioid withdrawal syndrome on health horizons

**DOI:** 10.3389/fgene.2023.1292148

**Published:** 2024-01-04

**Authors:** Uppala Radhakrishna, Senthilkumar Sadhasivam, Rupa Radhakrishnan, Ariadna Forray, Srinivas B. Muvvala, Raghu P. Metpally, Saumya Patel, Rakesh M. Rawal, Sangeetha Vishweswaraiah, Ray O. Bahado-Singh, Swapan K. Nath

**Affiliations:** ^1^ Department of Anesthesiology and Perioperative Medicine, University of Pittsburgh, Pittsburgh, PA, United States; ^2^ Department of Obstetrics and Gynecology, Corewell Health William Beaumont University Hospital, Royal Oak, MI, United States; ^3^ Department of Radiology and Imaging Sciences, Indiana University School of Medicine, Indianapolis, IN, United States; ^4^ Department of Psychiatry, Yale School of Medicine, New Haven, CT, United States; ^5^ Department of Molecular and Functional Genomics, Geisinger, Danville, PA, United States; ^6^ Department of Botany, Bioinformatics and Climate Change Impacts Management, School of Science, Gujarat University, Ahmedabad, India; ^7^ Department of Life Sciences, School of Sciences, Gujarat University, Ahmedabad, India; ^8^ Arthritis and Clinical Immunology Program, Oklahoma Medical Research Foundation, Oklahoma City, OK, United States

**Keywords:** cytochromes, biomarker, opioid use, neonatal opioid withdrawal syndrome CYP19A1, *CYP1A2*, *CYP4V2*, *CYP1B1*, *CYP24A1*

## Abstract

**Background:** Neonatal opioid withdrawal syndrome (NOWS), arises due to increased opioid use during pregnancy. Cytochrome P450 (CYP) enzymes play a pivotal role in metabolizing a wide range of substances in the human body, including opioids, other drugs, toxins, and endogenous compounds. The association between CYP gene methylation and opioid effects is unexplored and it could offer promising insights.

**Objective:** To investigate the impact of prenatal opioid exposure on disrupted CYPs in infants and their anticipated long-term clinical implications.

**Study Design:** DNA methylation levels of CYP genes were analyzed in a cohort of 96 placental tissues using Illumina Infinium MethylationEPIC (850 k) BeadChips. This involved three groups of placental tissues: 32 from mothers with infants exposed to opioids prenatally requiring pharmacologic treatment for NOWS, 32 from mothers with prenatally opioid-exposed infants not needing NOWS treatment, and 32 from unexposed control mothers.

**Results:** The study identified 20 significantly differentially methylated CpG sites associated with 17 distinct CYP genes, with 14 CpGs showing reduced methylation across 14 genes (*CYP19A1, CYP1A2, CYP4V2, CYP1B1, CYP24A1, CYP26B1, CYP26C1, CYP2C18, CYP2C9, CYP2U1, CYP39A1, CYP2R1, CYP4Z1, CYP2D7P1 and*), while 8 exhibited hypermethylation (*CYP51A1, CYP26B1, CYP2R1, CYP2U1, CYP4X1, CYP1A2, CYP2W1,* and *CYP4V2*). Genes such as *CYP1A2, CYP26B1, CYP2R1, CYP2U1,* and *CYP4V2* exhibited both increased and decreased methylation. These genes are crucial for metabolizing eicosanoids, fatty acids, drugs, and diverse substances.

**Conclusion:** The study identified profound methylation changes in multiple CYP genes in the placental tissues relevant to NOWS. This suggests that disruption of DNA methylation patterns in CYP transcripts might play a role in NOWS and may serve as valuable biomarkers, suggesting a future pathway for personalized treatment. Further research is needed to confirm these findings and explore their potential for diagnosis and treatment.

## Introduction

In the United States, opioid use disorder (OUD) is on the rise among women of reproductive age and is a major health problem. Infants exposed to opioids during pregnancy are likely to develop a drug withdrawal syndrome known as neonatal opioid withdrawal (NOWS) also called neonatal abstinence syndrome (NAS). NOWS incidence in the United States varies between 1.5 and 8.0 for every 1,000 hospital births and has increased by 433% from 2004 to 2014 (Jilani et al., 2019). Treatment of infants with NOWS often requires prolonged hospitalization in neonatal intensive care units (NICUs) for drug withdrawal treatment, but there is no effective preventative treatment. Symptoms of withdrawal most commonly appear within the first 24–48 h after birth, depending on the type of opioid exposure; however, not all infants exposed to opioids during pregnancy experience withdrawal or require medication treatment for withdrawal symptoms. Determining the type and level of care an infant exposed to opioids will need and the risk factors for developing NOWS, remain unanswered clinical questions.

One of the main barriers to the development of an effective NOWS treatment plan is due to the lack of biomarker(s) to help predict which infants will develop NOWS, as well as our incomplete understanding of the pathogenesis of this syndrome. Currently, neonatologists face a challenge in assessing the severity of NOWS in infants, as there is a dearth of research on the molecular aspects of this condition, including algorithms, genetics, and epigenetics. This hinders understanding of the underlying issues in infants exposed to opioids before birth, further exacerbated by the absence of FDA-approved medications for NOWS treatment. The diagnosis is currently based on a history of opioid exposure *in utero* and symptoms in the newborn consistent with opioid withdrawal. While there are two tests approved by the FDA at present, which measure drug concentration in urine and meconium (Gray et al., 2009; Gray et al., 2010), confirmation of exposure to opioids alone cannot help determine the risk of developing NOWS and the need for treatment.

DNA methylation is an epigenetic modification where a methyl group (CH3) is added to DNA cytosine bases, typically at CpG sites. DNA methyltransferase enzymes catalyze this process, and these changes can be either inherited or acquired during one’s lifetime. This modification silences genes at promoters, influencing tissue-specific regulation, development, and genomic stability. Aberrant DNA methylation is linked to diseases, notably cancer, and DNA demethylation via TET enzymes also impacts gene regulation and development (Schuebel et al., 2016). Pharmacoepigenetics pertains to genetic variations in pharmacogenetics caused by epigenetic modifications influencing an individual’s response to medical treatments (Gomez and Ingelman-Sundberg, 2009).

Numerous factors during pregnancy impact fetal growth, organ development, and overall health (Li et al., 2019; Metpally et al., 2019). These encompass nutrition, stress, toxins, smoking, addiction, and infections. The presence of these toxins can impact the placenta and modify gene expression (Radhakrishna et al., 2021a), potentially increasing the likelihood of future health risks as altered epigenetics shape later life outcomes (Radhakrishna et al., 2023).

Cytochrome P450 (CYPs) enzymes play a critical role in the metabolism of many medications (Lynch and Price, 2007). CYP enzymes are involved in opioid metabolism, however, the rate of opioid metabolism varies widely among individuals, and some have much faster metabolisms of drugs than others (Smith, 2009). The CYP enzymes can be inhibited or induced by drugs, such that inhibition reduces metabolism and induction increases it (Bibi, 2008). Their activity is also affected by factors such as age, gender, environment, lifestyle (smoking, alcohol, and opioids) genetic and epigenetic variations (Lin and Lu, 2001; Rostami-Hodjegan et al., 2004).

It has been reported that neonates with severe NOWS, exposed to methadone prenatally displayed higher levels of homozygosity for *CYP2B6* alleles at the 516 and 785 genetic loci (Mactier et al., 2017). Buprenorphine is an opioid that is approved by the FDA for treating opioid use disorder, acute pain, and chronic pain (Spreen et al., 2022). Buprenorphine is converted into its active metabolite, norbuprenorphine, by *CYP3A*, and SNPs in this gene may help predict the severity of buprenorphine-induced NOWS.

This study utilized a genome-wide methylation analysis to identify novel dysregulated CYPs in the placentas of mothers with opioid use disorder (OUD). These newly discovered CYPs could serve as potential markers to stratify the risk of opioid-exposed infants developing symptoms of NOWS before the symptoms become apparent. Gene ontology and pathway enrichment analyses were also conducted on the significant differentially methylated genes to gain deeper insights into their biological significance.

## Materials and methods

The research study was approved by the Institutional Review Board of Beaumont Health System, Royal Oak, MI, United States (HIC#: 2019-086). Pregnant women were retrospectively identified by chart review from William Beaumont Hospital, Royal Oak, MI. Informed consent was waived for this study because it exclusively involved the collection of discarded placental tissues from the subjects, in addition to obtaining limited de-identified basic demographic data from the hospital medical records. We collected demographic and clinical-pathological data, including age, sex, ethnicity, gestational age, and detailed information on drug exposure, as described in our previously published work (Radhakrishna et al., 2021b). Patients with OUD were diagnosed using the Diagnostic and Statistical Manual of Mental Disorders, Fifth Edition, or DSM-5, assessment criteria (Hasin et al., 2013).

A total of 96 formalin-fixed, paraffin-embedded (FFPE) placental tissue biopsies were very carefully collected and processed. The placental tissue samples were divided into 3 groups. Group-1 consisted of 32 prenatally opioid-exposed newborns that required treatment for NOWS (+Opioids/+NOWS), Group-2 had 32 prenatally opioid-exposed newborns that did not require treatment for NOWS (+Opioids/-NOWS), and Group-3 was the control group of newborns with no opioid exposure *in utero* and no NOWS (-Opioids/-NOWS, control). Neonatologists examined newborns diagnosed with NOWS using the clinical criteria specified in the ICD-10 under code P96.1. Infants born to mothers with a history of opioid or illicit drug use were monitored in the inpatient unit for 4–5 days to observe for signs of NOWS. The infant was scored using the Finnegan Neonatal Abstinence Scoring Tool (FNAST). This scoring was done by the *postpartum* nurses and/or NICU nurses. If the scores met the criteria for pharmacologic treatment, the baby was transferred to the NICU for further monitoring, scoring, and treatment. Parent involvement was encouraged to optimize non-pharmacologic treatment as the mainstay before and during and treatment course whether the baby is getting pharmacologic treatment or not. The Finnegan Neonatal Abstinence Scoring Tool (FNAST) was used to determine when the pharmacologic management with morphine should be initiated.

A previous publication detailed the utilization of Illumina methylationEPIC 850 k arrays (Illumina, Inc., San Diego, CA, United States). These arrays encompass over 850,000 individual CpG sites across the entire genome, offering single-nucleotide precision. Moreover, the arrays incorporate various cytochrome P450 genes (Radhakrishna et al., 2021b). We retrieved differentially methylated CG dinucleotide data from previously unpublished DNA methylation data of CYP-encoding genes (Radhakrishna et al., 2021b). From those, CG dinucleotides met several criteria: 1) at least a 5% difference in methylation level between prenatally opioid-exposed cases and controls, 2) FDR-corrected *p*-value <0.05, and 3) no overlap with single nucleotide polymorphisms to avoid potential confounding factors, were selected.

Then, to confirm the possibility of altered expression of CYP-encoding genes due to the differential methylation of the promoter region. A detailed account of sample selection, diagnosis, and analysis was explained in previously published work (Radhakrishna et al., 2021b).

### Statistical and bioinformatic analysis

Data (IDAT files) were normalized using Genome Studio software functional normalization and determined Cytosine methylation levels (ß-value) for each CpG site. Before analysis, we have removed all CpG-probes that have missing ß-values. Differential methylation was assessed by comparing the ß-values for the cytosine at each CpG locus in NOWS versus controls. To avoid confounding factors, we have removed probes associated with sex-chromosomes, non-specific probes, and probes targeting CpG sites within 10 bp of SNPs (each of which listed dbSNP entries within 10 bp of the CpG site) (Liu et al., 2013; Wilhelm-Benartzi et al., 2013; Zhang et al., 2013), further, SNPs with a minor allele frequency ≤ 0.05 were only considered for forwarding analysis.

Significantly differently methylated CpG sites between NOWS and controls were defined based on preset cutoff criteria FDR *p* < 0.05. Multiple CpG sites within a gene were resolved by the selection of the CpG with the highest AUC ROC ranking and the lowest *p*-value. The *p*-value for methylation differences between case and control groups at each locus was calculated as previously described (Vishweswaraiah et al., 2019; Radhakrishna et al., 2021b). Raw and FDR *p*-values corrected for multiple testing (Benjamini–Hochberg test) were calculated. Area under the receiver operating characteristic (AUC-ROC) AUC for combinations of loci was calculated using the ‘R’ program “ROCR” package (v3.5.0), based on methylation levels at the most significantly differently methylated CpG loci.

### Biological functional enrichment analysis

The most used annotation database in enrichment Gene Ontology (GO), and Kyoto Encyclopedia of Genes and Genomes (KEGG) pathway enrichment analyses were conducted with CpG methylation changes associated with NOWS with a statistical significance of *p*-value <0.05.

### Network interaction analysis using STRING

**FIGURE 1:**
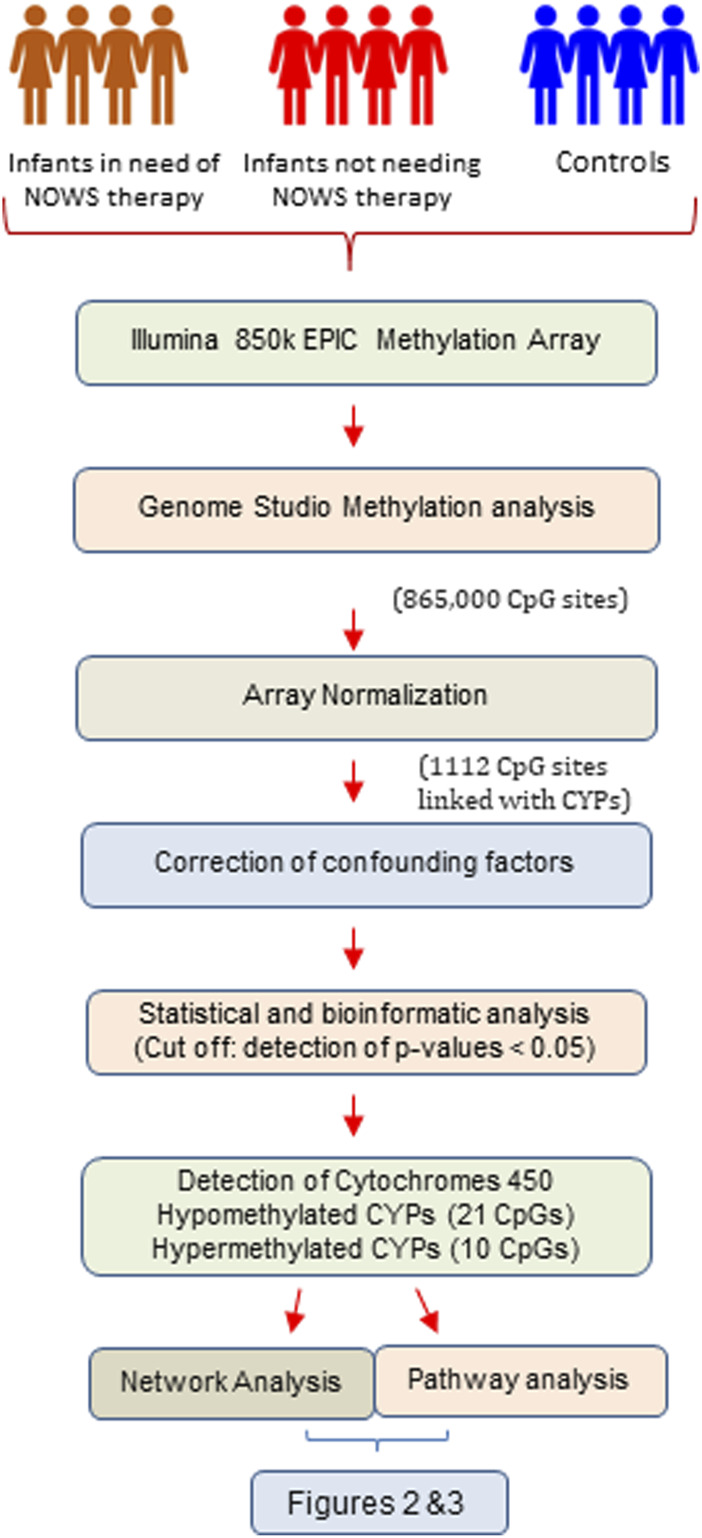
The flow chart illustrates the systematic workflow of genome-wide DNA methylation analysis.

Following the identification of 16 crucial genes from the methylome results, a comprehensive investigation was conducted to examine potential interactions among these genes. The tool employed for this purpose was the STRING network analysis, a well-established bioinformatics resource for mapping possible interactions between genes. A non-functional cytochrome P450 pseudogene, *CYP2D7P1*, was excluded from the analysis. The remaining list of 16 genes of interest, which demonstrated significant hypermethylation or hypomethylation changes in response to prenatal opioid exposure, were inputted into the STRING database (http://string-db.org) using a multiple protein search. This network was structured with a medium interaction score threshold of 0.40. Interaction sources such as text mining, experiments, databases, co-expression, and co-occurrence were selected for their unique contributions. Text mining and co-occurrence offer indirect associations from literature and an evolutionary perspective, experiments and databases contribute direct, known interactions, while co-expression discovers functionally linked genes through shared expression patterns (Figure 1).

## Results

The demographic characteristics of the NOWS and control groups were compared, and no significant differences were found. We have identified a total of 1,112 CpGs exclusively associated with CYPs across all four configuration studies after array normalization but before the removal of probes associated with confounding factors (Supplementary Tables S1–S4). The analysis performed with four configurations revealed CpGs with *p*-values <0.05 as 316, 222, 305, and 169 in analysis-1 (+Opioids/+NOWS versus + Opioids/-NOWS); analysis-2 (+Opioids/+NOWS), + (+Opioids/-NOWS), versus (-Opioids/-NOWS, control); analysis-3 (+Opioids/+NOWS), versus (-Opioids/-NOWS, control); and analysis-4 (+Opioids/-NOWS), versus (-Opioids/-NOWS, control), respectively. Additionally, the dataset comprised CpGs with FDR *p*-values <0.05, signifying significant differential methylation.

There were 20 CpG sites with FDR *p*-values <0.05, showing methylation differences of ±0.05 associated with 17 distinct CYP genes. Among these, 14 CpGs displayed hypomethylation across genes (*CYP19A1, CYP1A2, CYP4V2, CYP1B1, CYP24A1, CYP26B1, CYP26C1, CYP2C18, CYP2C9, CYP2U1, CYP39A1, CYP2R1, CYP4Z1,* and *CYP2D7P1*), while eight exhibited hypermethylation (*CYP51A1, CYP1A2, CYP26B1, CYP2R1, CYP2U1, CYP4X1, CYP2W1*, and *CYP4V2*). Notably, genes such as *CYP1A2, CYP26B1, CYP2R1, CYP2U1*, and *CYP4V2* exhibited both hyper and hypomethylation Tables 1–4).

**TABLE 1:** Detailed overview of CYP genes methylated status and their biological functions/associated diseases. Differentially methylated CpG markers are provided, with information about genes, the corresponding methylated CpG sites chromosome location, AUC, and the percentage of difference in methylation for each CpG and its biological significance based on FDR adjusted-p values <0.05 provided with (AUC) ≥0.75 for NOWS detection. (Hypo: Hypumethylation; Hyper: Hypermethylation; TSS200 denotes -200 base pairs to transcription start site; TSS1500 denotes -1500 base pairs to transcription start site). The analysis of +Opioids/+NOWS versus + Opioids/-NOWS. Significantly differentially methylated CYPs in NOWS.

CpG target ID	CYPs	CHR	p-Val	FDR p-Val	% Methylation			AUC	CI		Methyl status	CYP location
					Cases	Control	Change		Lower	Upper		
cg24775120	CYP2U1	4q25	2.1369E-16	1.8484E-10	59.05	70.04	−10.99	0.78	0.67	0.90	Hypo	Body
cg03544918	CYP2R1	11p15.2	1.1480E-12	9.9301E-07	17.26	25.57	−8.30	0.64	0.50	0.77	Hypo	TSS200
cg10896827	CYP4V2	4q35.1-q35.2	1.3299E-10	0.0001150	68.78	76.54	−7.76	0.81	0.70	0.92	Hypo	3′UTR
cg02143877	CYP24A1	20q13.2	2.2255E-10	0.0001925	46.88	56.26	−9.39	0.71	0.58	0.83	Hypo	1stExon
cg07225966	CYP26C1	10q23.33	1.5721E-09	0.0013599	46.63	55.59	−8.96	0.69	0.56	0.82	Hypo	TSS1500
cg24182584	CYP1A2	15q24.1	2.9321E-09	0.0025363	77.80	83.75	−5.95	0.70	0.57	0.83	Hypo	Body
cg12932492	CYP19A1	15q21.	1.1162E-08	0.0096552	45.42	53.95	−8.53	0.70	0.58	0.83	Hypo	5′UTR

**TABLE 2:** Detailed overview of CYP genes methylated status and their biological functions/associated diseases. Differentially methylated CpG markers are provided, with information about genes, the corresponding methylated CpG sites chromosome location, AUC, and the percentage of difference in methylation for each CpG and its biological significance based on FDR adjusted-p values <0.05 provided with (AUC) ≥0.75 for NOWS detection. (Hypo: Hypumethylation; Hyper: Hypermethylation; TSS200 denotes -200 base pairs to transcription start site; TSS1500 denotes -1500 base pairs to transcription start site). Analysis of (+Opioids/+NOWS), + (+Opioids/-NOWS), versus (-Opioids/-NOWS, control). Details of CpG targets significantly differentially methylated CYPs NOWS.

CpG target ID	CYPs	CHR	p-Val	FDR p-Val	% Methylation			AUC	CI		Methyl status	CYP location
					Cases	Control	Change		Lower	Upper		
cg23276445	CYP4Z1	1p33	7.4256E-16	6.5717E-10	10.47	17.38	−6.91	0.62	0.50	0.75	Hypo	Body
cg18244289	CYP26B1	2p13.2	3.6125E-13	3.1970E-07	7.50	13.07	−5.57	0.61	0.48	0.73	Hypo	TSS1500
cg24804666	CYP2C9	10q23.33	5.2321E-12	4.6304E-06	36.77	45.59	−8.82	0.65	0.53	0.77	Hypo	Body
cg14447606	CYP26B1	2p13.2	1.1869E-11	1.0504E-05	22.78	16.64	6.13	0.72	0.61	0.83	Hyper	Body
cg25217269	CYP39A1	6p12.3	1.9508E-11	1.7264E-05	29.90	38.17	−8.27	0.73	0.62	0.84	Hypo	5′UTR
cg12850546	CYP2D7P1	22q13.2	1.0507E-10	9.2989E-05	83.60	88.17	−4.57	0.72	0.60	0.83	Hypo	Body
cg09345077	CYP2C18	10q23.33	1.4402E-09	1.2746E-03	26.60	33.85	−7.26	0.59	0.46	0.71	Hypo	Body
cg18256630	CYP1A2	15q24.1	2.1520E-09	1.9045E-03	56.39	49.14	7.25	0.73	0.62	0.84	Hyper	TSS1500

**TABLE 3:** Detailed overview of CYP genes methylated status and their biological functions/associated diseases. Differentially methylated CpG markers are provided, with information about genes, the corresponding methylated CpG sites chromosome location, AUC, and the percentage of difference in methylation for each CpG and its biological significance based on FDR adjusted-p values <0.05 provided with (AUC) ≥0.75 for NOWS detection (Hypo: Hypumethylation; Hyper: Hypermethylation; TSS200 denotes -200 base pairs to transcription start site; TSS1500 denotes -1500 base pairs to transcription start site). Analysis of (+Opioids/+NOWS), versus (-Opioids/-NOWS, control). Details of CpG targets significantly differentially methylated CYPs NOWS.

CpG target ID	CYPs	CHR	*p*-Value	FDR p-Val	% Methylation			AUC	CI		Methyl status	CYP location
					Cases	Control	Change		Lower	Upper		
cg24804666	CYP2C9	10q23.33	9.6499E-16	8.5401E-10	34.06	45.59	−11.53	0.67	0.54	0.81	Hypo	Body
cg23276445	CYP4Z1	1p33	1.6696E-10	1.4776E-04	11.06	17.38	−6.33	0.60	0.46	0.74	Hypo	Body
cg18244289	CYP26B1	2p13.2	2.4461E-10	2.1648E-04	7.58	13.07	−5.49	0.59	0.45	0.73	Hypo	TSS1500
cg25217269	CYP39A1	6p12.3	2.8291E-09	2.5037E-03	29.73	38.17	−8.44	0.71	0.59	0.84	Hypo	Body
cg20646556	CYP2W1	7p22.3	3.9557E-09	3.5008E-03	52.26	44.09	8.17	0.67	0.54	0.80	Hypo	TSS200
cg07641350	CYP51A1	7q21.2	4.5592E-09	4.0349E-03	57.04	48.95	8.08	0.75	0.63	0.87	Hyper	5′UTR; Body
cg09799983	CYP1B1	2p22.2	4.2722E-08	3.7809E-02	40.69	48.98	−8.29	0.63	0.50	0.77	Hypo	Body

**TABLE 4:** Detailed overview of CYP genes methylated status and their biological functions/associated diseases. Differentially methylated CpG markers are provided, with information about genes, the corresponding methylated CpG sites chromosome location, AUC, and the percentage of difference in methylation for each CpG and its biological significance based on FDR adjusted-p values <0.05 provided with (AUC) ≥0.75 for NOWS detection (Hypo: Hypumethylation; Hyper: Hypermethylation; TSS200 denotes -200 base pairs to transcription start site; TSS1500 denotes -1500 base pairs to transcription start site). Analysis of (+Opioids/-NOWS), versus (-Opioids/-NOWS, control) with details of CpG targets significantly differentially methylated CYPs.

CpG target ID	CYPs	CHR	p-Val	FDR p-Val	% Methylation			AUC	CI		Methyl status	CYP location
					Cases	Control	Change		Lower	Upper		
cg00455178	CYP2R1	11p15.2	3.7555E-38	3.2485E-32	24.40	16.18	8.21	0.60	0.46	0.74	Hyper	TSS1500
cg23276445	CYP4Z1	1p33	1.3764E-14	1.1906E-08	10.03	17.38	−7.35	0.65	0.51	0.78	Hypo	Body
cg18256630	CYP1A2	15q24.1	7.4230E-13	6.4209E-07	58.68	49.14	9.55	0.78	0.66	0.89	Hyper	TSS1500
cg10896827	CYP4V2	4q35.1-q35.2	9.2815E-11	8.0285E-05	79.08	72.17	6.91	0.70	0.57	0.83	Hyper	3′UTR
cg14447606	CYP26B1	2p13.2	2.0154E-10	1.7433E-04	23.45	16.64	6.81	0.78	0.67	0.89	Hyper	Body
cg18244289	CYP26B1	2p13.2	2.3346E-10	2.0195E-04	7.59	13.07	−5.48	0.62	0.49	0.76	Hypo	TSS1500
cg24775120	CYP2U1	4q25	1.3274E-09	1.1482E-03	72.61	65.30	7.30	0.70	0.57	0.83	Hyper	Body
cg26051329	CYP4X1	1p33	5.5139E-09	4.7695E-03	67.21	59.68	7.53	0.76	0.64	0.88	Hyper	TSS1500
cg25217269	CYP39A1	6p12.3	3.5764E-08	3.0935E-02	30.30	38.17	−7.86	0.74	0.62	0.86	Hypo	5′UTR

### Gene interaction network analysis

**FIGURE 2:**
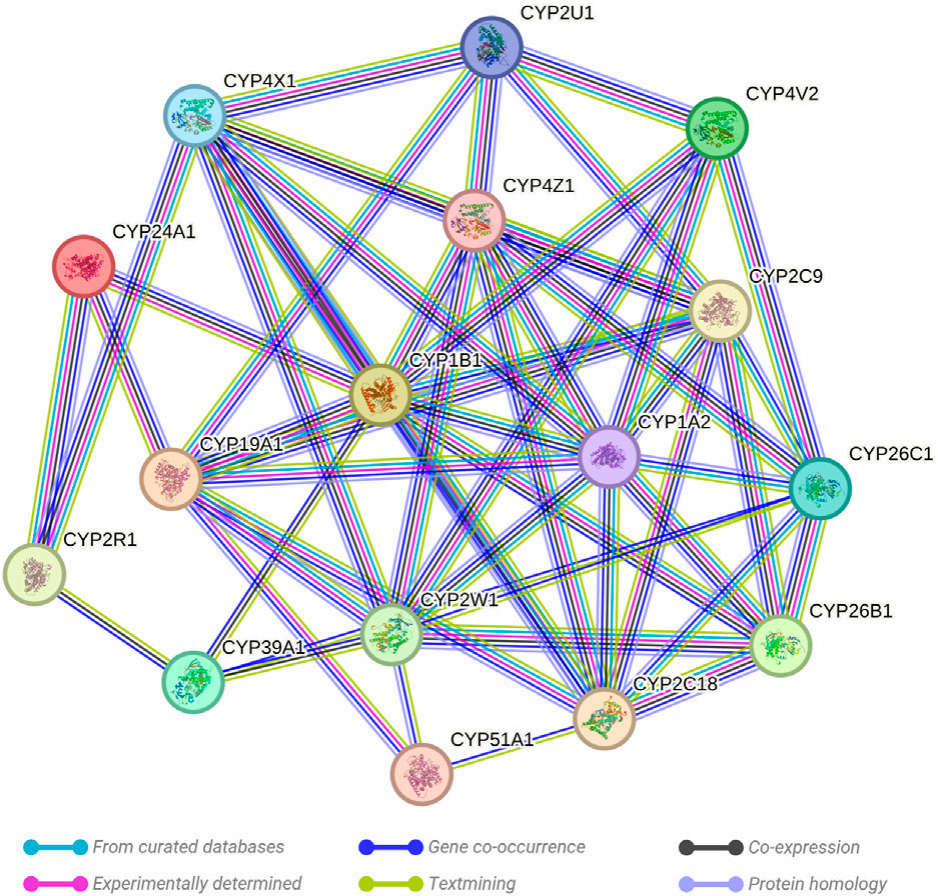
Evaluation of the links existing between the evaluated CYP genes. A critical assessment and integration of protein–protein was provided by the STRING database, including direct (physical) and indirect (functional) interactions. String predicted both physical and functional interactions between proteins. Number of nodes: 16, Number of edges: 54, Average node degree: 6.75, Avg. local clustering coefficient: 0.459, Expected number of edges: 1, and PPI enrichment *p*-value: <0.0001.

The STRING network analysis generated an interconnected network among the 16 key genes, presenting 54 edges in total. This network, visualized in Figure 2, exhibits the complex interplay between these genes. The network statistics displayed an average node degree of 6.75 and an average local clustering coefficient of 0.459. The expected number of edges was 1, which contrasted significantly with the observed results, confirming the proteins are indeed biologically interconnected as a group. This was further validated by the PPI enrichment *p*-value of <1.0e-16, suggesting that the interactions observed within this network significantly exceed random expectations. Analysis of individual genes highlighted their significance as central nodes in the network, demonstrating extensive interactions with numerous other genes. This indicates a key role of these genes within the network, particularly in their response to prenatal opioid exposure.

The findings offer insight into potential cooperative or antagonistic relationships among the selected genes. Changes in methylation status, either hypermethylation or hypomethylation, could impact the expression of these genes, thereby affecting metabolic and physiological responses to prenatal opioid exposure. These changes may further contribute to the development or severity of NOWS.

### Pathway and gene ontology analysis of dysregulated CYPs

To identify the KEGG pathway-associated genes and potential GO (Gene Ontology) classifications, terms related to biological processes (BP), molecular functions (MF), and signaling pathways were analyzed. As a result of the pathway analysis, 5 major dysregulated pathways associated with NOWS were identified.

Table 5 illustrates the functional enrichment analysis of the complex network, including some of the following pathways that were over-represented: hsa01100: Metabolic pathways, hsa05204: Chemical carcinogenesis - DNA adducts, hsa00100: Steroid biosynthesis, hsa00140: Steroid hormone biosynthesis, hsa00980: Metabolism of xenobiotics by cytochrome P450, and hsa04726: Serotonergic synapse. The biological processes (BPs) of the 16 dysregulated CYPs focused predominantly on the xenobiotic metabolic process, sterol metabolic process, vitamin metabolic process, organic acid metabolic process, omega-hydroxylase P450 pathway, epoxygenase P450 pathway, retinoic acid catabolic process, estrogen metabolic process, retinol metabolic process, aflatoxin metabolic process and fatty acid omega-oxidation as shown in Table 6. Regarding the cellular components (CCs), the study revealed a significant connection between these dysregulated CYPs and the endoplasmic reticulum membrane, as well as intracellular membrane-bound organelles, as demonstrated in Table 7. Additionally, Table 8 provided enriched molecular functions (MF) associated with the 16 differentially methylated CYPs. These functions encompassed iron ion binding, heme binding, monooxygenase activity, oxidoreductase activity, steroid hydroxylase activity, aromatase activity, caffeine oxidase activity, retinoic acid 4-hydroxylase activity, arachidonic acid 14,15-epoxygenase activity, arachidonic acid epoxygenase activity, and cytochrome P450 fatty acid omega-hydroxylase activity, along with estrogen 16-alpha-hydroxylase activity.

**TABLE 5:** Gene ontology (GO) terms associated with differentially methylated genes that are associated with NOWS such as KEGG pathway enrichment for the 12 CYP genes with altered methylation patterns linked to NOWS.

Term	*p*-value	Genes	FDR
hsa00830:Retinol metabolism	3.289E-08	CYP2C9, CYP2W1, CYP26B1, CYP26C1, CYP1A2, CYP2C18	3.947E-07
hsa01100: Metabolic pathways	5.768E-06	CYP2C9, CYP2W1, CYP26B1, CYP24A1, CYP26C1, CYP2U1, CYP2R1, CYP1A2, CYP51A1, CYP19A1, CYP2C18	3.461E-05
hsa05204: Chemical carcinogenesis - DNA adducts	0.0001400	CYP2C9, CYP1A2, CYP1B1, CYP2C18	0.0005600
hsa00100: Steroid biosynthesis	0.0003960	CYP24A1, CYP2R1, CYP51A1	0.0011879
hsa00140: Steroid hormone biosynthesis	0.0038021	CYP1A2, CYP1B1, CYP19A1	0.0091251
hsa00980: Metabolism of xenobiotics by cytochrome P450	0.0059561	CYP2C9, CYP1A2, CYP1B1	0.0119122
hsa04726: Serotonergic synapse	0.0125972	CYP2C9, CYP4X1, CYP2C18	0.0215952

**TABLE 6:** Gene ontology (GO) terms associated with differentially methylated genes that are associated with NOWS such as Biological processes (BP) enrichment analysis in NOWS.

Term	*p*-value	Genes	FDR
GO: 0016125∼sterol metabolic process	4.915E-15	CYP39A1, CYP26B1, CYP26C1, CYP4V2, CYP51A1, CYP1B1, CYP19A1	6.341E-13
GO: 0006805∼xenobiotic metabolic process	1.555E-12	CYP2C9, CYP2W1, CYP26B1, CYP2U1, CYP2R1, CYP1A2, CYP1B1, CYP2C18	1.003E-10
GO: 0006082∼organic acid metabolic process	1.045E-08	CYP2C9, CYP2W1, CYP2U1, CYP2R1, CYP2C18	4.173E-07
GO: 0006766∼vitamin metabolic process	1.294E-08	CYP26B1, CYP24A1, CYP26C1, CYP2R1	4.173E-07
GO: 0097267∼omega-hydroxylase P450 pathway	8.115E-08	CYP2C9, CYP2U1, CYP1A2,CYP1B1	2.094E-06
GO: 0019373∼epoxygenase P450 pathway	5.654E-07	CYP2C9, CYP1A2, CYP1B1, CYP2C18	1.216E-05
GO: 0034653∼retinoic acid catabolic process	5.537E-06	CYP2W1, CYP26B1, CYP26C1	0.0001020
GO: 0008210∼estrogen metabolic process	0.0002887	CYP2C9, CYP1A2, CYP1B1	0.0046559
GO: 0042572∼retinol metabolic process	0.0008042	CYP1A2, CYP1B1, CYP2C18	0.0115263
GO: 0046222∼aflatoxin metabolic process	0.0038481	CYP2W1, CYP1A2	0.0496408
GO: 0010430∼fatty acid omega-oxidation	0.0053835	CYP24A1, CYP4V2	0.0578726

**TABLE 7:** Gene ontology (GO) terms associated with differentially methylated genes that are associated with NOWS such as Cellular Component (CC) enrichment analysis.

Term	*p*-value	Genes	FDR
GO: 0005789∼endoplasmic reticulum membrane	2.9986E-17	CYP51A1, CYP19A1, YP2C18, CYP39A1, CYP4Z1, CYP2C9, CYP2W1, CYP26B1, CYP4X1, CYP26C1, CYP2U1, CYP4V2, CYP2R1, CYP1A2, CYP1B1	3.89818E-16
GO: 0043231∼intracellular membrane-bounded organelle	3.07657E-06	CYP39A1, CYP2C9, CYP2W1, CYP2U1, CYP2R1, CYP1A2, CYP1B1, CYP2C18	1.99977E-05

**TABLE 8:** Gene ontology (GO) terms associated with differentially methylated genes that are associated with NOWS such as KEGG pathway enrichment for the 12 CYP genes with altered methylation patterns linked to NOWS.

Term	PValue	Genes	FDR
GO: 0005506∼iron ion binding	8.401E-33	CYP51A1, CYP19A1, CYP2C18, CYP39A1, CYP4Z1, CYP2C9, CYP2W1, CYP26B1, CYP4X1, CYP24A1, CYP26C1, CYP2U1, CYP4V2, CYP2R1, CYP1A2, CYP1B1	3.444E-31
GO: 0020037∼heme binding	2.401E-32	CYP51A1, CYP19A1, CYP2C18, CYP39A1, CYP4Z1, CYP2C9, CYP2W1, CYP26B1, CYP4X1, CYP24A1, CYP26C1, CYP2U1, CYP4V2, CYP2R1, CYP1A2, CYP1B1	4.923E-31
GO: 0004497∼monooxygenase activity	5.083E-27	CYP51A1, CYP19A1, CYP2C18, CYP39A1, CYP2C9, CYP2W1, CYP26B1, CYP26C1, CYP2U1, CYP4V2, CYP2R1, CYP1A2, CYP1B1	6.947E-26
GO: 0016705∼oxidoreductase activity, acting on paired donors, with incorporation or reduction of molecular oxygen	1.605E-22	CYP39A1, CYP2C9, CYP2W1, CYP26B1, CYP26C1, CYP4V2, CYP2R1, CYP51A1, CYP1B1, CYP19A1, CYP2C18	1.645E-21
GO: 0016712∼oxidoreductase activity, acting on paired donors, with incorporation or reduction of molecular oxygen, reduced flavin or flavoprotein as one donor, and incorporation of one atom of oxygen	1.259E-17	CYP2C9, CYP2W1, CYP2U1, CYP2R1, CYP1A2, CYP1B1, CYP19A1, CYP2C18	1.033E-16
GO: 0070330∼aromatase activity	8.457E-14	CYP4Z1, CYP2C9, CYP26C1, CYP1A2, CYP1B1, CYP19A1, CYP2C18	5.779E-13
GO: 0008395∼steroid hydroxylase activity	1.027E-13	CYP39A1, CYP2C9, CYP2W1, CYP2U1, CYP2R1, CYP19A1, CYP2C18	6.017E-13
GO:0008401∼retinoic acid 4-hydroxylase activity	4.779E-08	CYP2W1, CYP26B1, CYP26C1, CYP2C18	2.449E-07
GO: 0016491∼oxidoreductase activity	2.719E-05	CYP2C9, CYP26B1, CYP26C1, CYP1A2, CYP51A1	0.0001239
GO: 0001972∼retinoic acid binding	0.0001099	CYP2W1, CYP26B1, CYP26C1	0.0004506
GO: 0008404∼arachidonic acid 14,15-epoxygenase activity	0.0023697	CYP4Z1, CYP2C9	0.0088324
GO: 0034875∼caffeine oxidase activity	0.0031584	CYP2C9, CYP1A2	0.0107912
GO: 0102033∼cytochrome P450 fatty acid omega-hydroxylase activity	0.0063075	CYP2U1, CYP4V2	0.0184719
GO: 0101020∼estrogen 16-alpha-hydroxylase activity	0.0063075	CYP1A2, CYP1B1	0.0184719
GO: 0008392∼arachidonic acid epoxygenase activity	0.0149196	CYP2C9, CYP2C18	0.0407803
GO: 0019825∼oxygen binding	0.0288621	CYP19A1, CYP2C18	0.0739592

## Discussion

Currently, there is no effective way to diagnose and treat NOWS before it occurs. This is partially due to asymptomatic manifestation in NOWS infants because not all infants require pharmacotherapy, as well as its complicated molecular mechanisms involved in the long-term effects. Infants with severe symptoms of NOWS have a greater risk of adverse long-term developmental outcomes including lower IQ, poor educational testing performance, lower attention, meeting disability criteria, and requiring additional classroom services when compared to children with prenatal opioid exposure who did not develop NOWS or healthy controls (Oei et al., 2017; Fill et al., 2018). A significant challenge is distinguishing the neurobiological effects of opioid agonists from various other confounding factors, including tobacco, alcohol, nonmedical drugs, environmental influences, and additional medical risks (Logan et al., 2013; Radhakrishnan et al., 2022a; Radhakrishnan et al., 2022b). The effects of opioids during pregnancy extend beyond nociceptive processes to include effects on gastrointestinal, endocrine, and autonomic functions as well as cognition, behavior, and reward circuitry for the mother and developing fetus, and subsequently the offspring (Radhakrishnan et al., 2021; Radhakrishnan et al., 2022a; Radhakrishnan et al., 2022b; Radhakrishnan et al., 2022c; Vishnubhotla et al., 2022; Wise et al., 2023).

Through diverse comparative analyses, the study identified 20 CpG sites with significant differences in their methylation patterns. These studies aimed to unravel the epigenetic and molecular dysregulation associated with NOWS. The investigations employed various methods, including the following approaches. An overview of CYP enzyme substrates, inhibitors, and inducers with clinical significance in NOWS patients and controls is provided in Table 9.

**TABLE 9:** Significant Cytochrome P450 Enzymes and Their Inhibitors, Inducers, and Substrates.

Gene	Substrate	Inhibitors	Inducer	Modulator	Antagonist	Methyl
CYP1A2	Betaxolol, Ropinirole, Theophylline, Disopyramide, Ropivacaine, Zolmitriptan, Acetaminophen, Amitriptyline, Methadone, Olanzapine, Chlorzoxazone, Clozapine, Mirtazapine, Palonosetron, Tacrine, Triamterene, Sorafenib, Zolpidem, Trimethoprim, Loratadine, Nabumetone, Quinine, Chlorpromazine, Flutamide, Haloperidol, Erlotinib, Mephenytoin, Nortriptyline, Fluorouracil, Cinnarizine, Propranolol, Fenfluramine, Clonidine, Diclofenac, Imatinib, Guanabenz, Estrone, Verapamil, Tamoxifen, Warfarin, Tizanidine, Norethisterone, Riluzole, Clopidogrel, Malathion, Etoposide, Naproxen, Pentoxifylline, Trifluoperazine, Perphenazine, Dacarbazine, Terbinafine, Ethanol, Cyclobenzaprine, Maprotiline, Azelastine, Ethinylestradiol, Ramelteon, Glycopyrronium, Azathioprine, Frovatriptan, Levobupivacaine, Cinacalcet, Methoxyflurane, Praziquantel, Melatonin, Benzocaine, Hesperetin, Leflunomide, Pimozide, Carvedilol, Doxepin, Cilostazol, Domperidone, Dexfenfluramine, Flecainide, Aminophylline, Metoclopramide, Clomipramine, Dasatinib, Lumiracoxib, Oxtriphylline, Rasagiline, Theobromine, Acenocoumarol, Aminophenazone, Antipyrine, Fenethylline, Bromazepam, Thiothixene, Etoricoxib, 8-azaguanine, Xanthine, 9-Methylguanine, Phenacetin, Hypoxanthine, 9-Deazaguanine, Estrone sulfate, Flunarizine, Febuxostat, Lorcaserin, Bicifadine, Lofexidine, Pirfenidone, Apremilast, GTS-21, Lumateperone, Mianserin, Eltrombopag, Asenapine, Vadimezan, Dapagliflozin, Lonafarnib, Pazopanib, Agomelatine, Apixaban, Axitinib, Bendamustine, Benzyl alcohol, (R)-warfarin, Ulipristal, Perampanel, Pomalidomide, Tasimelteon, Zotepine, Tegafur, Doxofylline, Propacetamol, Ramosetron, Ixazomib, Selumetinib, Icotinib, Istradefylline, Binimetinib, Voxilaprevir, Deutetrabenazine, Lisofylline, Nicotine, Primaquine, Resveratrol, Capsaicin, Fluvoxamine, Bortezomib, Caffeine, Anagrelide, Lidocaine, Grepafloxacin, Mexiletine, Promazine, Imipramine, Fluoxetine, Duloxetine, Rofecoxib, Efavirenz, Paroxetine, Thiabendazole, Zileuton, Estradiol, Mefenamic acid, Ranitidine, Ondansetron, Alosetron, Lomefloxacin, Selegiline, Tocainide, Nifedipine, Propafenone, Temafloxacin, Lopinavir, Genistein, Stiripentol, Abametapir, Triclabendazole, Dihydralazine, Enasidenib, Estradiol acetate, Estradiol benzoate, Estradiol cypionate, Estradiol valerate, Methylene blue, Fexinidazole, Rucaparib	Ticlopidine, Citalopram, Moxifloxacin, Nevirapine, Valproic acid, Ethambutol, Remoxipride, Rosiglitazone, Nitric Oxide, Enoxacin, Pefloxacin, Cimetidine, Ciprofloxacin, Zafirlukast, Methoxsalen, Dexmedetomidine, Trovafloxacin, Tranylcypromine, Methimazole, Nalidixic acid, Rosoxacin, Propofol, Cinoxacin, Quinidine, Famotidine, Isoniazid, Ketoconazole, Gatifloxacin, Norfloxacin, Atazanavir, Tegaserod, Amiodarone, Gemifloxacin, Ofloxacin, Orphenadrine, Sparfloxacin, Anastrozole, Gemfibrozil, Mibefradil, Deferasirox, Quercetin, Abiraterone, Simeprevir, Safinamide, Besifloxacin, Niclosamide, Sulconazole, Vemurafenib, Dabrafenib, Flumequine, Cannabidiol, Lenvatinib, Zucapsaicin, Dosulepin, Viloxazine, Lobeglitazone, Sarafloxacin, Curcumin, Pazufloxacin, Capmatinib, Osilodrostat, Prulifloxacin, Diacerein, Abemaciclib, Brincidofovir, Clascoterone, Rhein, Obeticholic acid, Menadione, Carbamazepine, Rabeprazole, Midostaurin, Fluvoxamine, Bortezomib, Caffeine, Anagrelide, Lidocaine, Grepafloxacin, Mexiletine, Promazine, Imipramine, Fluoxetine, Duloxetine, Rofecoxib, Efavirenz, Paroxetine, Thiabendazole, Zileuton, Estradiol, Mefenamic acid, Ranitidine, Ondansetron, Alosetron, Lomefloxacin, Selegiline, Tocainide, Nifedipine, Propafenone, Temafloxacin, Lopinavir, Genistein, Stiripentol, Abametapir, Triclabendazole, Dihydralazine, Enasidenib, Estradiol acetate, Estradiol benzoate, Estradiol cypionate, Estradiol valerate, Methylene blue, Fexinidazole, Rucaparib	Phenytoin, Omeprazole, Phenylephrine, Griseofulvin, Secobarbital, Streptozocin, Meperidine, Ritonavir, Albendazole, Nafcillin, Modafinil, Primidone, Rifampicin, Phenobarbital, Fosphenytoin, Belinostat, Dovitinib, Armodafinil, beta-Naphthoflavone, Teriflunomide, Osimertinib, Menadione, Carbamazepine, Rabeprazole, Midostaurin, Nicotine, Primaquine, Resveratrol, Capsaicin			Hyper
						Hypo
CYP24A1	25-hydroxyvitamin D3 (calcidiol)	Ketoconazole, Imatinib, 25-hydroxyvitamin D3 analogs, 1,25-dihydroxyvitamin D3 analogs.	Calcium and phosphate, PTH (parathyroid hormone) Interleukin-1β (IL-1β) and other cytokines:			Hypo
CYP26B1	Retinoic acid	R115866 (also known as liarozole)	Retinoic acid			Hypo
						Hyper
CYP2C18	Phenytoin	Fluconazole, Fluoxetine, Sulfaphenazole:	Rifampin, Barbiturates, St. John's Wort (Hypericum perforatum): A herbal supplement			Hypo
CYP2C9	Amitriptyline, Azilsartan, Capecitabine, Celecoxib, Clopidogrel, Diclofenac, Doxepin, Fluoxetine, Fluvastatin, Glibenclamide, Glimepiride, Glipizide, Glyburide, Ibuprofen, Irbesartan, Lesinurad, Lornoxicam, Losartan, Meloxicam, Nateglinide, Olodaterol, Ospemifene, Phenytoin, Piroxicam, Rosiglitazone, Ruxolitinib, S-Naproxen, S-Warfarin, Suprofen, Tamoxifen, Tolbutamide, Torsemide, Valproic Acid, Venlafaxine, Voriconazole, Zafirlukast.	Amiodarone, Capecitabine, Ceritinib, Efavirenz, Fenofibrate, Fluconazole, Fluvastatin, Fluvoxamine, Isoniazid, Metronidazole, Phenylbutazone, Quercetin, Rucaparib, Sulfamethoxazole, Sulfaphenazole, Voriconazole, Zafirlukast	Carbamazepine, Dabrafenib, Enzalutamide, Nevirapine, Phenobarbital, Rifampin, Secobarbital, St. John's Wort			Hypo
CYP2R1	Vitamin D3 (cholecalciferol)					Hypo
						Hyper
CYP2U1	Retinoids,					Hypo
						Hyper
CYP39A1	Oxysterols 24-hydroxycholesterol, 25-hydroxycholesterol and 27-hydroxycholesterol, cholesterol					Hypo
CYP4V2	Arachidonic acid, Eicosapentaenoic acid, docosahexaenoic acid	Fenofibrate,Voriconazole, Ketoconazole	Peroxisome proliferator-activated receptor (PPAR) agonists,			Hyper
		Diltiazem				Hypo
CYP4X1	Arachidonic acid, linoleic acid.	17-octadecynoic acid, Lithospermum erythrorhizon	aryl hydrocarbon receptor (AhR) and the pregnane X receptor (PXR),			Hyper
CYP4Z1	Retinoids and steroids,		Dexamethasone, Progesterone			Hypo
CYP51A1	Lanosterol	fluconazole, itraconazole, voriconazole, Posaconazole, Terbinafine	Rifampin			Hyper
CYP1B1	Caffeine, Theophylline, Progesterone, Flutamide, Erlotinib, Amodiaquine, Testosterone, Estrone, Estradiol, Dasatinib, Triclabendazole, Estradiol acetate, Estradiol benzoate, Estradiol cypionate, Estradiol valerate, Tamoxifen, Melatonin	Menadione, Daunorubicin, Propofol, Ketoconazole, Mitoxantrone, Paclitaxel, Resveratrol, Cannabidiol, Doxorubicin, Tamoxifen, Melatonin	Biotin, Omeprazole, Betamethasone, Lansoprazole, Prednisone, Hydrocortisone, Methylprednisolone, Isoprenaline, Primaquine, udesonide, Triclocarban, oxorubicin			Hypo
CYP19A1	Methadone, Testosterone, Edetic acid, Stanolone, Methyltestosterone, Betamethasone	Nicotine, Troglitazone, Diethylstilbestrol, Mefloquine, Aminoglutethimide,Carbimazole, Raloxifene, Tamoxifen, Drostanolone, Etretinate, Testolactone, Letrozole, Exemestane, Tioconazole, Ketoconazole, Melatonin, Miconazole, Econazole, Anastrozole, Paclitaxel, Danazol, Naringenin, Bifonazole, Cyproterne acetate, Norgestrel	Chlorotrianisene, Chlorphenesin, Prasterone sulfate, Sulfathiazole, Nandrolone decanoate	Edetate calcium disodium anhydrous, Edetate disodium anhydrous	Letrozole	Hypo
CYP26C1	Retinoic acid (RA).	Talarozole, ketoconazole, Fluconazole, Azolyl retinoids				Hypo
CYP2W1	Benzothiazole GW610, Fluorobenzothiazole 5F203(Phortress), Duocarmycin analogs ICT2705, ICT2706, Farnesol, Geranylgeraniol, Benzphetamine, Indoles, indolines	Retinoic acid	Imatinib, 5-Aza-2'-deoxycytidine (DAC), Linoleic acid (LA)			Hyper

### Distinguishing NOWS from prenatal opioid exposure without NOWS

This comparison aims to identify the epigenetic and molecular differences between newborns with NOWS requiring treatment, and infants exposed to opioids prenatally without withdrawal symptoms and those not requiring treatment for NOWS. This analysis identifies factors leading to withdrawal in specific infants (Table 1).

### Distinguishing prenatal opioid abuse versus normal controls (OUD detection)

This comparison focuses on identifying epigenetic markers associated with maternal opioid abuse during pregnancy. We analyzed to identify the differences between infants born to mothers with opioid use disorder (OUD) and infants born to mothers without substance abuse issues. Comprehending these variances aids in identifying prenatal opioid exposure and evaluating the impact of maternal opioid abuse on the newborn’s molecular and epigenetic traits (Table 2).

### Distinguishing NOWS versus unexposed controls

In this comparison, we examined the epigenetic alterations in newborns with NOWS in comparison to infants born to mothers who did not use opioids during pregnancy. Through this analysis, we pinpointed NOWS-related epigenetic changes, offering crucial insights into the mechanisms driving withdrawal symptoms in newborns (Table 3).

### Distinguishing opioid-induced epigenetic changes

This comparison studied the epigenetic changes in newborns who were exposed to opioids during prenatal development in comparison with infants born to mothers who refrained from opioid use during pregnancy. We discovered opioid-induced epigenetic changes, offering crucial insights into the molecular mechanisms influenced by early exposure to opioids (Table 4).

**FIGURE 3:**
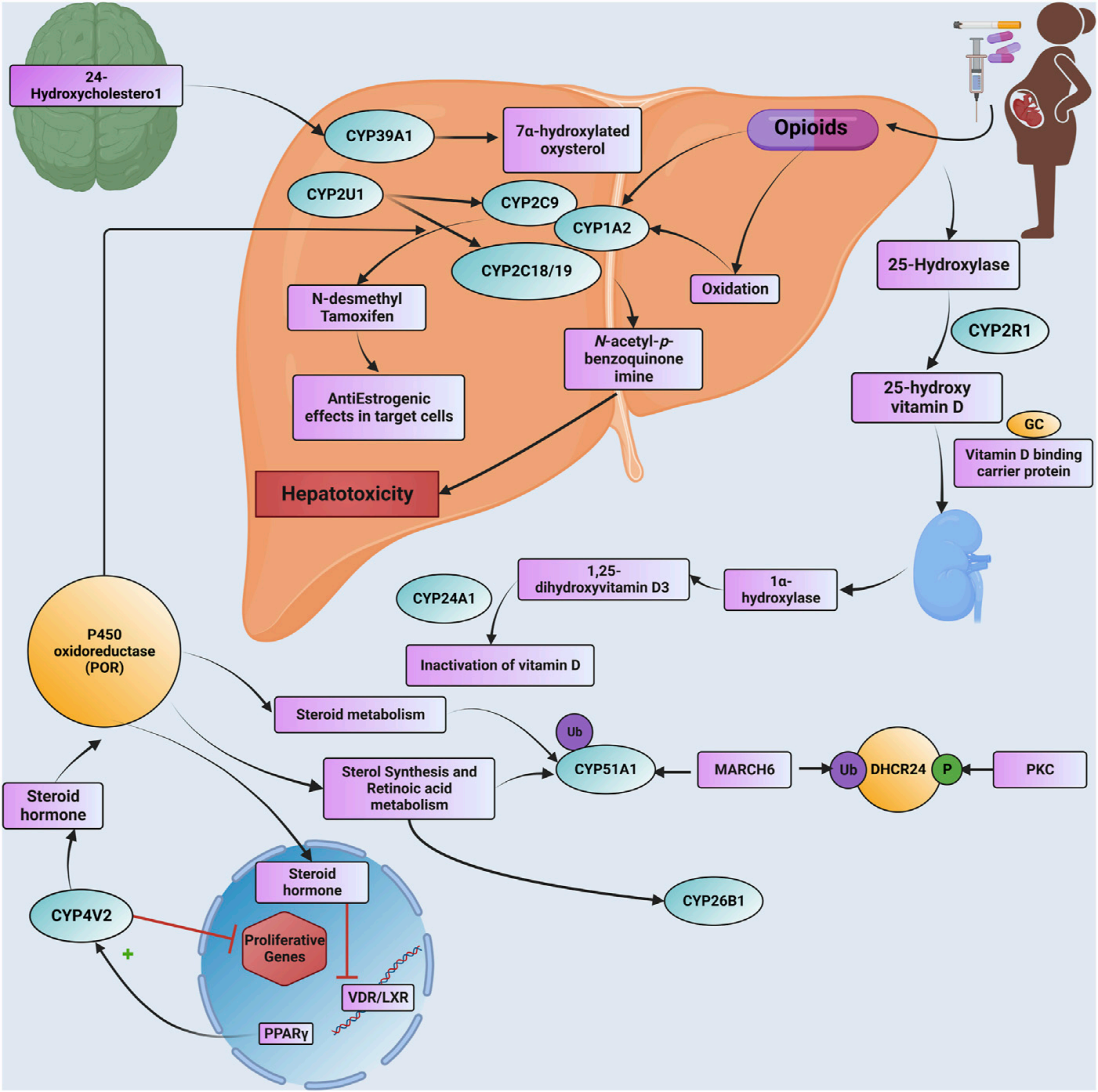
A detailed Schematic illustration is provided of how epigenetic modifications influence drug metabolism and transport through CYP genes, including specific information about dysregulated CYP enzymes. Additionally, the text outlines the pathogenetic events associated with NOWS and highlights specific CYP genes that undergo significant methylation. Abbreviations. GC: GROUP-SPECIFIC COMPONENT; Ub: ubiquitination; P: phosphorylation; DHCR24: delta (24)-sterol reductase; MARCH6: Membrane Associated Ring-CH-Type Finger 6; PKC: Protein kinase C; PPARG: Peroxisome Proliferator-Activated Receptor Gamma; VDR: Vitamin D Receptor; LXR: Liver X Receptor

Drug metabolism by genetically polymorphic enzymes can have significant clinical implications relating to drug toxicity, therapeutic failure, drug-drug interactions, disease susceptibility, abuse liability as well and the number of drugs consumed by people who take drugs. The present study reveals that 20 CpGs align with 17 distinct CYPs, each showcasing unique methylation patterns. Figure 3 illustrates how epigenetic regulation affects drug metabolism and transport with details of dysregulated CYPs.

Depending on circumstances, CYP-active drugs can inhibit, induce, or act as substrates for enzymes. Inhibition curbs metabolism, while induction enhances it (McDonnell and Dang, 2013). Methylation-driven disruptions in CYPs within the placenta may potentially contribute to various abnormalities observed in both NOWS and OUD cases. Insufficient data exists regarding the enduring impacts of maternal opioid usage during pregnancy on infants diagnosed with NOWS. Intermittent anomalies arising from dysregulated CYPs in infants experiencing NOWS may not manifest immediately, but could instead become noticeable later in life. These conditions encompass ailments such as Non-alcoholic Fatty Liver Disease (NAFLD), type 2 diabetes, cardiovascular diseases, and various psychiatric disorders, among others. To explore this hypothesis, we require extended follow-up studies.

### CYP1A2

*CYP1A2* is primarily expressed in the liver (Zanger and Schwab, 2013), but it has also been detected in the brain, suggesting a potential role in protecting the brain from xenobiotics (Yun et al., 1998). Additionally, *CYP1A2* plays a critical role in converting chemical pollutants from cigarette smoke into carcinogenic compounds (Taşçıoğlu et al., 2021b). Individuals who smoke tend to be more prone to self-report opioid use disorders in comparison to non-smokers (Young-Wolff et al., 2017). Notably, a link has been established between the genetic variation rs762551 in the *CYP1A2* gene and an increased susceptibility to lung cancer among smokers (Taşçıoğlu et al., 2021a), Moreover, a noteworthy correlation has emerged connecting *CYP1A2* with drug addiction (Zhang et al., 2016).

### CYP4V2

*CYP4V2* is a key enzyme that is associated with NAFLD, a significant health concern affecting 30% of adults in the United States (Garduno and Wu, 2021). NAFLD is marked by dysbiosis, a condition impacting non-alcohol consumers, and is characterized by the accumulation of fat, liver inflammation, fibrosis, and cirrhosis. Opioid use is more common in NAFLD with cirrhosis, high BMI, and psychiatric disorders (Moon et al., 2021).

### CYP51A1

The *CYP51A1* in humans, also known as lanosterol 14α-demethylase is the most evolutionarily conserved member of the CYP450 (Rezen et al., 2004), and is pivotal in hepatic cholesterol synthesis (Lewinska et al., 2013). *CYP51A1* removes two methyl groups from lanosterol during oxidative reactions. Cholesterol is essential for life, but elevated serum cholesterol levels raise the risk of conditions like high blood pressure, type 2 diabetes, coronary heart disease (CHD), stroke, and cardiovascular disease (CVD). It has been reported that opioid use and cholesterol levels are related since cholesterol levels affect opioid signaling in cell models (Zheng et al., 2012; Kazemi et al., 2021). Preterm birth, affecting 20%–40% of infants exposed to opioids during pregnancy, poses a substantial risk factor for NOWS (Allocco et al., 2016; Sheikh et al., 2016a). A previous study found that CYP51A1 gene variations are associated with preterm birth among women (Sheikh et al., 2016b).

### CYP24A1

The *CYP24A1* gene encodes a 24-hydroxylase enzyme that regulates active vitamin D levels in the body by converting the active form, 1,25-dihydroxy vitamin D3 or calcitriol, into an inactive form when it is no longer required (Prosser and Jones, 2004). Opioid addiction and ultraviolet ray addiction (Kemeny et al., 2021), occur more frequently in individuals with insufficient vitamin D levels, suggesting a potential role for this vitamin in neurodevelopment and safeguarding dopaminergic pathways in the adult brain (Eserian, 2013), Additionally, vitamin D is vital for preventing neonatal hypoxic-ischemic brain damage (Anjum et al., 2018). This vitamin is further strongly known for its role in cognitive impairment, dementia, psychosis, and autism (Weller et al., 2021). Transcription from the human *CYP24A1* gene is increased by calcium ions and by excess amounts of 1,25-dihydroxy vitamin D3 (Nebert and McKinnon, 1994; Norman et al., 2001). Variations in the *CYP24A1* gene lead to an accumulation of vitamin D3 and an associated hypervitaminosis D phenotype (Griffin et al., 2020), Hypervitaminosis D is linked to a range of severe health hazards, including reduced appetite, constipation, depressive symptoms, and memory impairment—similar issues experienced by individuals with chronic opioid use (Nelson and Camilleri, 2015).

### CYP39A1

The endoplasmic reticulum protein, oxysterol 7-α-hydroxylase 2 encoded by *CYP39A1*, is pivotal in converting cholesterol to bile acids. It is primarily expressed in the liver and converts 24-hydroxycholesterol (24OHC) into a 7-alpha-hydroxylated form (Li-Hawkins et al., 2000). Aberrant *CYP39A1* expression may contribute to neurodegenerative diseases like Alzheimer’s (Matsuoka et al., 2020), however, its role in NOWS is not yet known. *CYP39A1* protein is involved in the pathway of cholesterol degradation, which is part of steroid metabolism (Stiles et al., 2014). Cholesterol is a structural component of membranes, as well as a precursor for bile acid and steroid hormone synthesis (Kenworthy, 2002). Individuals with OUD may experience concurrent mental health issues like depression and anxiety, which can impact their hormonal systems and stress responses.

### CYP26B1

The *CYP26A1, CYP26B1*, and *CYP26C1* enzymes are a group of enzymes that are highly conserved in vertebrates, metabolizing retinoic acid (RA), a vitamin A derivative, and potentially playing a role in xenobiotic metabolism. Vitamin A is vital for embryonic development, organ formation, immunity, and eye health (Bastos Maia et al., 2019). Currently, retinoids are used to treat a variety of skin conditions, and antiaging skincare, as well as slow the effects of photoaging (Szymanski et al., 2020), addiction, and abuse causing premature aging, wrinkles, and other skin problems (Inci et al., 2017). Furthermore, retinoic acid impacts the adult brain; animal studies have shown that isotretinoin (retinoids) inhibits neurogenesis in the hippocampus when administered (Bremner and McCaffery, 2008). Interestingly, isotretinoin induces apoptosis in different types of cells in the body and decreases the enzyme telomerase reverse transcriptase (*TERT*) activity (Das et al., 2022). Increased *TERT* expression restores telomerase activity, suggesting that transcriptional control may play a role in a wide array of diseases, including cancer and aging (Colebatch et al., 2019). Lack of Telomerase/TERT expression leads to progressive telomere erosion in dividing human cells, while critically shortened telomeres induce permanent growth arrest, increased incidence of diseases, and poor survival (Trybek et al., 2020). The impaired TERT could be another contributing factor, as opioid addicts typically have a much shorter lifespan than non-addicts (Smyth et al., 2007; Lewer et al., 2020). Yet, the life expectancy of an infant with NOWS remains unknown.

### CYP2U1

*CYP2U1* is expressed in the amygdala and prefrontal cortex of humans and is involved in brain and immune functions. *CYP2U1* is involved in cytokine production, long-chain fatty acid synthesis, drug absorption, and the metabolism of foreign substances and eicosanoids. However, alcohol consumption and smoking can impair its function. Amygdalae, integral components of the limbic system, have a significant role in memory, decision-making, and the processing of emotional responses, including fear, anxiety, and aggression (Amunts et al., 2005). There is a link between anxiety and drug addiction due to perturbations of the anxiety and fear centers in the brain (Brady et al., 2013).

### CYP2R1

*CYP2R1* is an enzyme involved in vitamin D biosynthesis that is significantly hypermethylated in this study. Deficiency in vitamin D may lead to many health conditions, including chronic pain, addiction to opioids (Kemeny et al., 2021), sleep disorders, and irritability (Archontogeorgis et al., 2018). Infants with NOWS are commonly seen with symptoms such as irritability, lethargy, muscle weakness, frequent respiratory problems, and decreased sleep (Patrick et al., 2020).

### *CYP4X1* and *CYP4Z1*

The hypomethylated *CYP4X1* and *CYP4Z1* genes together are presumed to be involved in human breast and ovarian cancers (Blahos et al., 1986; Savas et al., 2005; Murray et al., 2010). Chronic opioid use is more common among cancer patients, possibly because of confounding anxiety and/or depression that often accompany cancer diagnosis (Marcusa et al., 2017).

### CYP2C18

*CYP2C18* participates in the metabolism of retinoic acid processes (Thatcher and Isoherranen, 2009). Isotretinoin, a retinoid compound, is derived from Vitamin A. Reports indicate that employing synthetic vitamin A (retinoids) like isotretinoin (accutane) during pregnancy may result in miscarriage, premature birth, and an array of developmental abnormalities in the embryo and fetus (Ross et al., 2000). Opioid use during pregnancy is linked to poor fetal growth, preterm birth, stillbirth, and birth defects, as well as NOWS (Yazdy et al., 2015; Lind et al., 2017).

### CYP2C9

*CYP2C9* enzyme plays a very important role in the metabolism of vitamin K antagonist anticoagulants, such as S-warfarin, S-acenocoumarol, and phenprocoumon, commonly used to treat thromboembolic diseases (Daly and King, 2006; Chen et al., 2018). Smoking is prevalent among 95% of opioid users, and tobacco contains high levels of vitamin K (Chun et al., 2009). It has been reported that opioid users are at a greater risk of ischemic stroke (Lee et al., 2013). Another key gene, VKORC1, encodes the VKORC1 (vitamin K epoxide reductase) protein, which is a key enzyme in the vitamin K cycle and was also hypomethylated in the current study (Radhakrishna et al., 2021b).

### CYP1B1 and CYP19A1

The interplay of *CYP1B1* and *CYP19A1* in estrogen metabolism and biosynthesis is pivotal in understanding their impact on addiction development. *CYP1B1*, a key player in estrogen metabolism, facilitates the conversion of estrone (E1) and estradiol (E2) into diverse metabolites (Tsuchiya et al., 2004). Estrogen has an impact on critical neurotransmitter systems in the brain, specifically dopamine, serotonin, and opioids, which are intricately connected to the reward circuitry associated with the development of addiction (Krolick et al., 2018; Martinez et al., 2016). The aromatase enzyme, also known as CYP19A1, plays a crucial role in estrogen synthesis. *CYP19A1*-mediated alterations in estrogen levels play a significant role in influencing diverse responses to substances of abuse, potentially accentuating gender-related variations in vulnerability to addiction (Moran-Santa Maria et al., 2014).

### Study Limitations

Our study has several limitations. First and foremost, we require additional investigation into the role of the dysmethylated CpGs we’ve identified to establish whether changes in methylation at these CpG sites might impact gene transcription. Additionally, we lack prior addiction history information for women who used opioids during pregnancy. Another significant limitation of this study is that the diagnosis of NOWS predominantly depends on assessing clinical signs and symptoms in newborns right after birth. Nevertheless, numerous clinical manifestations stemming from CYP dysregulations may only surface later in a child’s growth and development. Consequently, linking these initial clinical indicators in infants to potential long-term consequences can pose a formidable challenge. Furthermore, it is important to note that methylation patterns are tissue-specific. Just because certain genes are methylated in the placenta does not necessarily imply the same methylation status in the liver of newborns, a crucial site for drug metabolism.

## Conclusion

In summary, our cohort study revealed dysregulation in several crucial CYP genes linked to NOWS. These genes are closely involved in the metabolism of drugs, vitamin K, vitamin D, vitamin A, and bile acids. Moreover, they are linked to various health conditions, such as Autism, Alzheimer’s disease, non-alcoholic fatty liver disease, and emotional-behavioral dysregulation in infants. The study has significant implications for understanding the long-term effects of CYP dysregulation. Nevertheless, further investigation is necessary to fully comprehend the underlying molecular mechanisms responsible for the observed CYP gene dysregulations following prenatal opioid exposure. This deeper understanding could potentially open doors for advancements in personalized medicine down the line. Further research is required to explore their feasibility, effectiveness, and integration with other relevant factors.

## Data Availability

The original contributions presented in the study are included in the article/Supplementary Material, further inquiries can be directed to the corresponding author.

## Supplementary material

SUPPLEMENTARY TABLE S1 The analysis of +Opioids/+NOWS versus +Opioids/-NOWS.

SUPPLEMENTARY TABLE S2 Analysis of (+Opioids/+NOWS), + (+Opioids/-NOWS), versus (-Opioids/-NOWS, control).

SUPPLEMENTARY TABLE S3 Analysis of (+Opioids/+NOWS), versus (-Opioids/-NOWS, control).

SUPPLEMENTARY TABLE S4 Analysis of (+Opioids/-NOWS), versus (-Opioids/-NOWS, control).
